# Development of an Educational Gamification Strategy to Enhance the Food Safety Practices of Family Farmers in Public Food Markets of Northeast Brazil: A Case Study

**DOI:** 10.3390/foods12101972

**Published:** 2023-05-12

**Authors:** Noádia Priscila Araújo Rodrigues, Mirella Silva de Morais, Nísia Carolina Damacena Bezerra, Erik Henrique Morais Pereira, Élcio Antônio Garcia Júnior, Jéssica Bezerra dos Santos Rodrigues, Sônia de Paula Alexandrino de Oliveira, Evandro Leite de Souza

**Affiliations:** 1 Laboratory of Didactic Restaurant and Beverages, Department of Gastronomy, Center for Technology and Regional Development, Federal University of Paraiba, João Pessoa 58051-900, PB, Brazil; npar@academico.ufpb.br (N.P.A.R.);; 2 Laboratory of Food Microbiology, Department of Nutrition, Health Sciences Center, Federal University of Paraiba, João Pessoa 58058-600, PB, Brazil

**Keywords:** food hygiene, food handlers, family farmer, game-based learning

## Abstract

This study aimed to develop an educational gamification strategy to enhance the food safety practices of family farmers in public food markets in a city in Northeastern Brazil (João Pessoa, PB, Brazil). A good manufacturing practices (GMP) checklist was used to verify hygienic-sanitary conditions in the food markets. Educational game tools addressing foodborne diseases and GMP with information about the prevention of foodborne diseases, good food handling practices, and safe food storage were developed. Pre- and post-training assessments were done to evaluate food handlers’ knowledge and food safety practices. Microbiological parameters of food samples were analyzed before and two months after the training. Results indicated unsatisfactory hygiene conditions in the examined food markets. There was a very strong positive correlation between “implementation of GMP” and “production and process controls” (R = 0.95; *p* ≤ 0.05) and between “production and process controls” and “hygiene habits of handlers” (R = 0.92; *p* ≤0.05). There was no homogeneity between answers before and after the training for the knowledge of family farmers regarding “prevention of foodborne diseases” and “safe food handling”. There were improvements in the measured microbiological parameters of foods sold by family farmers after the application of the developed educational gamification training. These results showed the developed educational game-based strategy as being effective in raising awareness of hygienic sanitary practices, helping to promote food safety, and reducing risks for the consumers of street foods at family farmers’ markets.

## 1. Introduction

The commercialization of food products derived from smallholder agriculture, predominantly consisting of family farmers, plays a significant role in reinforcing food security, which is achieved by providing consumers with access to locally sourced and fresh produce [1]. Family farmers play a pivotal role in ensuring food safety due to various aspects, such as the common use of limited pesticides and chemicals in the crop cultivation processes and reducing the risks of chemical residues in the food supply. Furthermore, family farmers play an important role in establishing robust relationships between farmers and consumers, fostering trust and transparency in the food supply chain, in addition to facilitating the creation of sustainable and equitable food systems and promoting accountability for food safety [2,3].

The United Nations Food and Agriculture Organization (FAO) highlights that small-scale and family farmers are responsible for producing up to 80% of the food consumed in Asia and sub-Saharan Africa, underscoring the indispensable role they play in ensuring food security [3,4,5]. There are around 16.5 million family farms in Latin America, where roughly 60 million people work. Approximately 55% of these family farms are in South America, and a significant part of these family farms are in Brazil [3]. In developing countries like Brazil, the food products sold by family farmers may be called street foods, being characterized as the sale of food and drink in a market or in other public spaces, such as street pavements or other informal arrangements [4,5]. Nevertheless, family farmers’ markets can also be considered a food safety risk due to the commonly reported low level of hygiene during the production and commercialization of processed food and the implication on foodborne disease outbreaks. On the other hand, there are government regulations for hygienic measures in handling food that impose certain requirements for food safety [6,7,8,9]. 

For instance, within the Brazilian territory, the Sanitary Surveillance Agency, commonly referred to as ANVISA, holds regulatory authority over establishments engaged in the production, distribution, and sale of food products. Additionally, foods sold by family farmers are also subject to compliance with food safety regulations set by ANVISA. These regulations stipulate adherence to a range of requirements, including the use of clean and properly sanitized utensils and equipment, appropriate storage of food, and adherence to proper hand hygiene practices by vendors. Moreover, these establishments must comply with regional and local regulations concerning food safety, which may encompass guidelines related to the handling of food, temperature control, packaging protocols, and training [10]. However, there is no requirement regarding the structure of how the training should be developed. Mainly for family farmers marketing street foods, whose issues related to hygiene are critical in many countries, there is an urgent demand to develop training strategies regarding food safety practices [6,7,8,9,11].

In developing countries, around 70% of diarrhea cases are associated with the consumption of street foods [12]. Studies have reported unsatisfactory microbiological characteristics in foods commercialized by family farmers, which have been linked to the non-adoption of food regulations and good manufacturing practices (GMPs) [9,13]. Foodborne diseases have been a global health concern, causing substantial morbidity and mortality and spending significant public resources [14,15]. Although the consumption of street food in family farmers’ markets may pose a potential risk of foodborne diseases, its cultural and socioeconomic significance is also recognized; it is estimated that around 2.5 billion people consume street food daily, including food sold in public food markets [5,12]. Studies have demonstrated that the public health risks of consuming street foods are linked to frequent non-compliance with GMPs, as well as limited knowledge of food safety by people handling street foods [7,12], which corroborates the urgent need to develop strategies to improve awareness and literacy about food safety and better hygiene practices by these handlers [7,14].

Training on food safety and hygiene with the adoption of GMP has shown a positive impact on the behavior and practices of food handlers, remaining as an initial step to ensure important knowledge related to food safety. However, there are several barriers to achieving proper food safety and hygiene training programs, such as appropriate adjustments according to the stage of the food supply chain and market and regional and cultural characteristics [14,16,17]. Training interventions could be planned considering international or national guidelines, legislations, and certifications, besides having the support of local governments and non-governmental initiatives [18]. The application of adequate training programs should lead to changes in food handling practices and improve food safety. Gamification learning is among the strategies for training applications, including the possibility of training in food safety practices [19,20,21]. This strategy could be evaluated regarding its efficacy in enhancing the food safety knowledge and practices of family farmers [19,20,21]. It is important that innovative and interactive educational gamification approaches are integrated into the qualification strategies of food handlers at all levels of the food supply chain, including family farmers’ markets. Educational gamification training strategies could help food handlers to effectively acquire and retain the necessary knowledge and skills needed to ensure food safety and promote the provision of high-quality food products to consumers [22,23].

This study aimed to develop an educational gamification strategy to enhance the awareness level about food safety practices of family farmers in public food markets located in a Brazilian Northeastern city, as well as their compliance with GMP.

## 2. Materials and Methods

### 2.1. Study Area and Participants

This study was conducted with family farmers marketing street foods in all public food markets (*n* = 9) in the city of João Pessoa, PB, Brazil (Northeast region). All study protocols were approved by the Ethics Committee on Human Research (Health Sciences Center, Federal University of Paraíba, Brazil, CAAE nº 56810722.9.0000.5188). Thirty percent of 75 family farmer groups working in this city were randomly selected and voluntarily agreed to participate in the study. Each group was formed with four to eight family farmers. Information related to sociodemographic factors, including gender, age, city origin, sold foodstuffs, and previous training in food safety was collected.

### 2.2. Assessment of GMP in Public Food Markets

The GMP assessment was carried out through visits to family farmers in public food markets before and after the implementation of the training program. A structured checklist was used to register the adoption of GMP [24]. This checklist was intended to assess the degree of compliance to the Brazilian GMP recommendations, which were developed using the principles recommended by the World Health Organization (WHO) [24]. The checklist was organized into four parts: (i) buildings and facilities, which listed requirements for the maintenance, layout, and operation of food processing facilities; (ii) production and process controls, which listed general sanitation processes and outlined requirements for storage and distribution; (iii) hygiene habits of handlers, which listed the general personal hygiene habits to be adopted by handlers; and (iv) implementation of the GMP manual, which listed the necessary controls to ensure the suitability of food produced for human consumption.

### 2.3. Assessment of Knowledge of Family Farmers Regarding Food Safety Practices

A pre-training test with 39 questions was given to the family farmers to assess their knowledge regarding food safety practices. After the training, a post-test was applied to assess the improvements in knowledge of food safety practices. The questions were grouped into topics about foodborne diseases, food safety handling, and food inspection and storage [25]. The participants were asked to circle the correct answer for each question and were given the choices of “true” or “false” [26].

### 2.4. Training of Family Farmers

The training modules were organized into (i) ways to prevent foodborne diseases, (ii) food safe handling, and (iii) safe food storage. The learning objectives of the training modules were to improve knowledge about personal hygiene, cross-contamination, environmental hygiene, food storage and process control, pest control, and cleaning and sanitation. Educational games were developed for each module, and each training application using games was made for groups of four to eight family farmers with a duration of 15 to 20 min on alternate days. The training was applied to family farmers who sold street foods in public food markets without interfering in their activities in the marketplaces.

**Figure 1:**
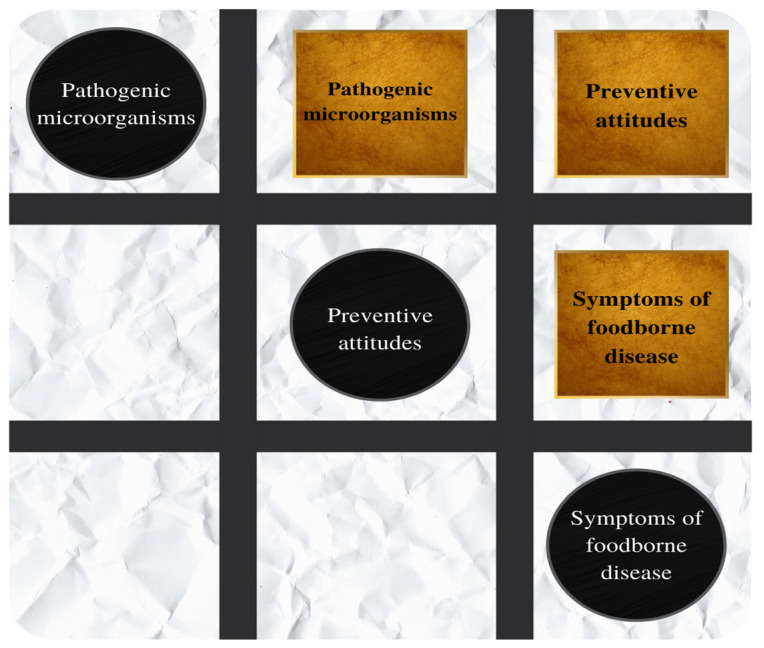
Banner for tic-tac-toe about foodborne diseases. The signs for the game are pathogenic microorganisms, attitudes toward prevention, and symptoms of foodborne diseases.

To train about ways to prevent foodborne diseases, a learning game was designed to simulate the tic-tac-toe game. The board game is shown in Figure 1. Square and circle signs showing information about pathogenic microorganisms involved in foodborne diseases, their symptoms, and measures to prevent them were used. These signs showed the main symptoms of each foodborne disease and measures to prevent contamination or reduce the etiological agents to safe levels. The foodborne disease etiological agents addressed were *Listeria monocytogenes*, *Salmonella* spp., *Staphylococcus aureus*, *Escherichia coli*, *Bacillus cereus*, and *Clostridium perfringens*. The participant who completed the triad of information regarding each etiological agent, the symptoms of the disease caused by each etiological agent, and the measures to prevent it was the winner.

**Figure 2:**
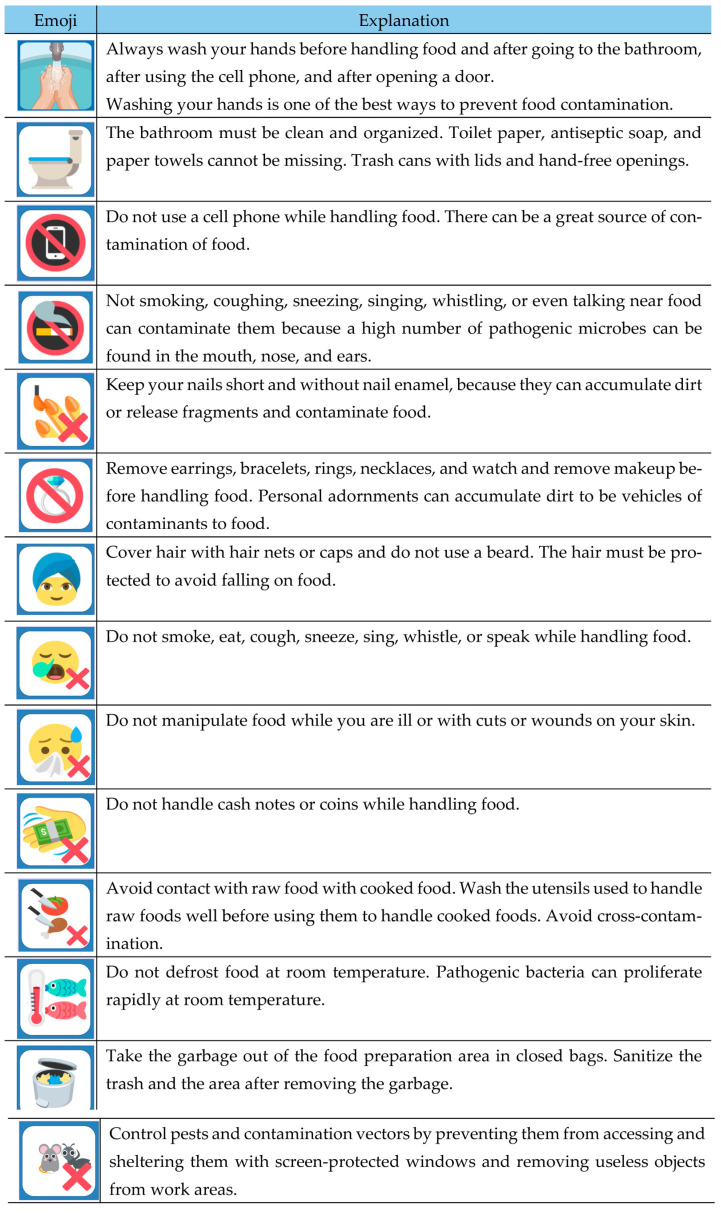
Material used in the educational game for training of GMP for food: emojis with the explanations used for GMP training.

To train about safe food handling for family farmers, a poster using emojis to convey the main concepts of the GMP of foods was developed (Figure 2). Different emojis were used to learn each subject covered in the training. The concepts discussed in the game were correct handwashing, use of personal protective equipment, ways to avoid cross-contamination, hygiene habits of food handlers, pest control, and safe food storage. The participants were asked to choose an image each time and listen to an explanation about it. 

**Figure 3:**
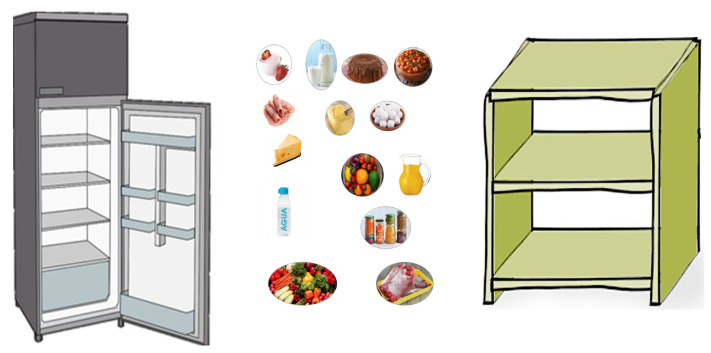
Material used in the educational game for safe food storage training: refrigerator, food cupboard, and foodstuffs to place in the correct storage place.

To train about safe food storage, a banner with a refrigerator image and a food cupboard image was developed. Several signs with different food images were printed. These different types of foods needed to be placed in the correct storage place, i.e., the refrigerator or the food cupboard. There were foodstuffs that needed to be placed in cold or frozen storage (these foods needed to be placed in the lower or upper part of the refrigerator, respectively) or stored at room temperature (these foods needed to be placed in the food cupboard). Topics about cleaning material that must be stored far away from foods and care with ready-to-eat foods, such as time and temperature control for safety, were also mentioned during the training application (Figure 3).

The training modules used for the participants were developed based on available literature regarding the Hazard Analysis and Critical Control Points (HACCP), guidelines for investigation and control of foodborne disease outbreaks, and the 2030 Agenda for Sustainable Development from Codex Alimentarius [27,28,29].

### 2.5. Microbiological Analysis of Foods Commercialized by Family Farmers in Public Food Markets

The food products most frequently traded in these establishments were baking products, cheeses, and fruit pulps. Samples of these products were collected in each public food market included in the study (*n* = 9) during pre- and post-training periods in three different batches. All food samples (*n* = 648) were analyzed for counts of total coliforms, *E. coli*, molds and yeasts, and the presence of *Salmonella* spp. Counts of coagulase-positive *Staphylococcus* were performed only in baking products and cheeses, the presumptive *B. cereus* counts were performed only in baking products, and the presence of *L. monocytogenes* was verified only in cheeses. The selection of these tests took into consideration the requirements set by the Brazilian regulations for food safety [24,30].

Twenty-five g of each food sample was homogenized with 225 mL of sterile peptone water (peptone 0.1 g/100 mL; HiMedia, Mumbai, India) with a stomacher (Marconi, Piracicaba, SP, Brazil) for 2 min (25 ± 0.5 °C) and serially diluted (1:9; 10^−2^–10^−5^, *v*/*v*) with sterile peptone water solution (0.01 g/100 mL). The microbiological analyses were conducted following standard procedures [30,31] using VRBG, EMB, PDA, and MYP agar (HiMedia) to count total coliforms, *E. coli*, molds and yeasts, and *B. cereus* (colony-forming units per g or mL-CFU/g or mL), respectively. Standard procedures [30,31,32] were used to verify the presence of *Salmonella* spp. and *L. monocytogenes* and count coagulase-positive staphylococci (CFU/g or mL) using XLD, BP, and CL (with OCLA supplement) agar (Oxoid, Hampshire, UK), respectively. Microbiological analyses were done in triplicate. The results were compared to the microbiological standards for the specific food groups as set by Brazilian regulations [30].

### 2.6. Data Processing and Statistical Analysis

The responses for both knowledge of food safety practices and GMP questions were coded on a two-point scale with “0” for an incorrect answer and “1” for every correct response. All correct answers were totaled and converted to a percentage score. Frequencies and descriptive statistics were used to summarize scores for results related to food safety knowledge, GMP, and microbiological analyses. The level of knowledge of family farmers and GMP adequacy were considered high for scores of ≥80% and low for scores of <80% [26]. The variables in the datasets were tested for normality distribution before performing further statistical analysis.

A Spearman’s correlation test was run to evaluate the relationship between the checked GMP aspects. A principal component analysis (PCA) was run to sort the public food markets according to the adequacy of checked GMP aspects. Non-parametric Friedman ANOVA with Durbin-Conover pairwise comparisons as follow-up analyses were used to compare the results of microbiological analyses before and after the training. Homogeneity chi-square tests were run to verify significant differences between the frequencies of responses to the questionnaire about knowledge of food safety practices administered before and after the training. A *p*-value of ≤0.05 was considered statistically significant. The statistical analysis was conducted using R software (Version 2.15.3, Ross Ihaka and Robert Gentleman, University of Auckland, Auckland, New Zealand).

## 3. Results

### 3.1. Sociodemographic Profile of Family Farmers and Diversity of Products Commercialized in Public Food Markets

**Figure 4:**
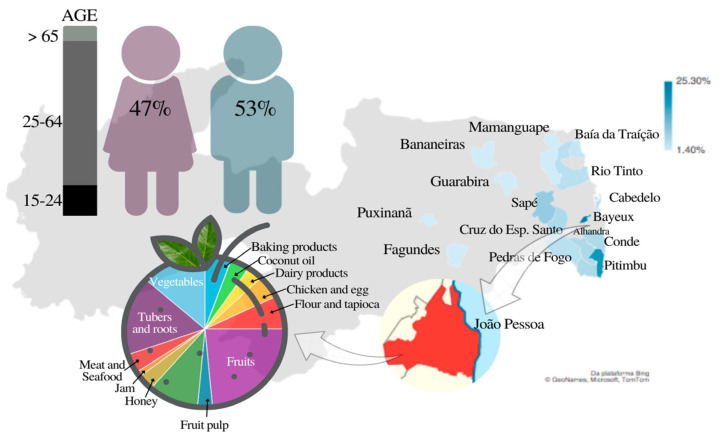
Map of Paraíba state (Brazil) showing the cities of origin of the family farmers included in the study who commercialize foods in public food markets in the city of João Pessoa (Paraíba, Brazil) and types of commercialized products.

The sociodemographic profile of the family farmers and the diversity of products commercialized by them in public food markets are shown in Figure 4. The ages and genders of 75 family farmer groups (572 people) and a map where places with blue color represent the cities of origin of the family farmers are presented. The darker the blue, the greater the concentration of the family farmers who came from that city to work at the nine public food markets located in the city of João Pessoa (red color).

Thirteen different food groups were commercialized by family farmers in the public food markets included in this study. Baking products (loaves, cookies, and cakes), cheeses, and fruit pulps were the most frequently commercialized foods.

Regarding the education level of the family farmers, the majority had a lower level of education (52.5%) with only elementary school completed, while 34.8% had completed high school. Additionally, the average income level of the family farmers was indicative of poverty or extreme poverty, with an average monthly income of approximately 850 reais, currently equivalent to roughly 200 dollars.

### 3.2. Assessment of GMP in Public Food Markets

**Figure 5:**
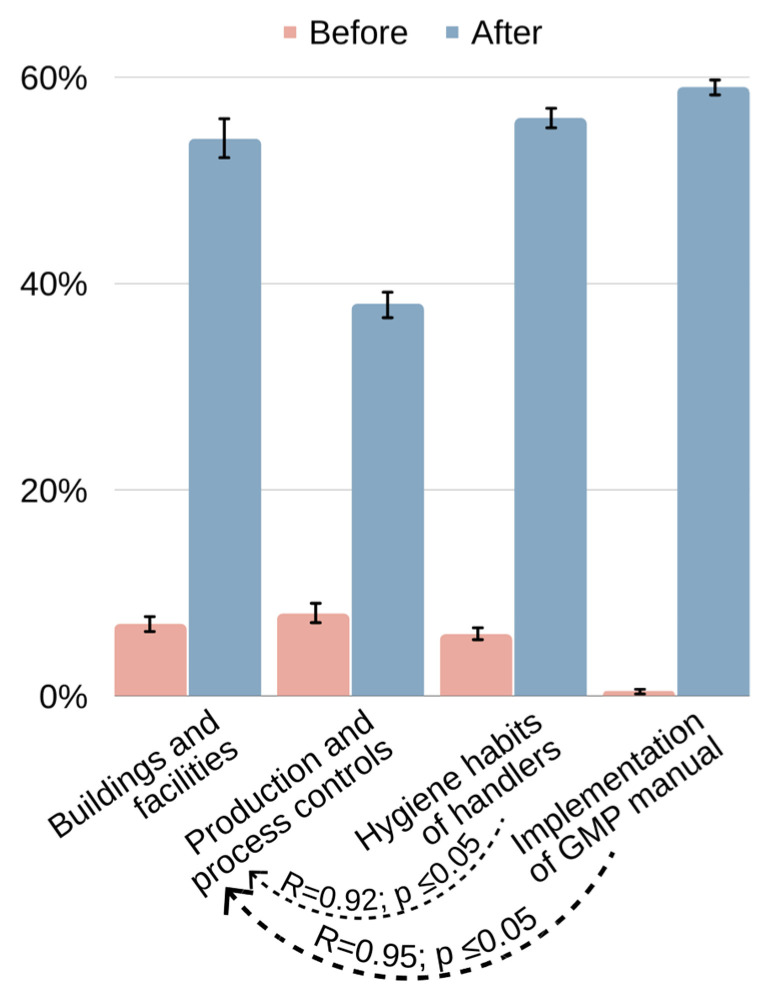
Bar graphs with significant correlations between the variables of the results of GMP assessment, namely, buildings and facilities, production and process controls, hygiene habits of handlers, and implementation of the GMP manual.

**Figure 6:**
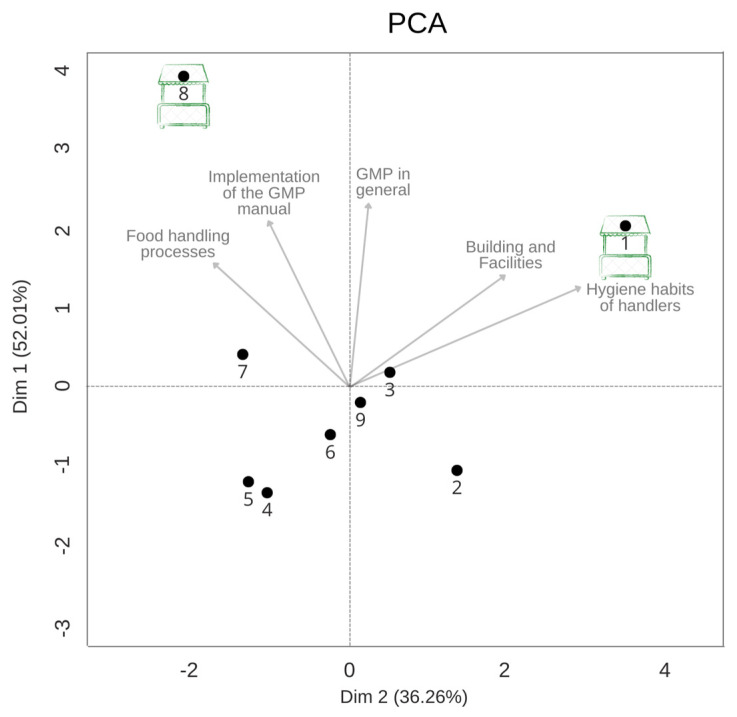
PCA plot ordainment graph of public food markets analyzed with parameters of good manufacturing practices, namely, buildings and facilities, production and process controls, hygiene habits of handlers, and implementation of the GMP manual.

Figure 5 and Figure 6 show the results of the GMP assessment in public food markets. Regarding the questions about “buildings and facilities,” the scores ranged from 7% to 54% adequacy. The lower scores were related to the external areas of these places, which were not free of rubble, weeds, or trash. Waste and disposal containers were generally found in inadequate numbers, and some were not fully covered. There were not enough sanitary facilities for all food handlers. There were few washbasins for food handling or distribution areas and a lack of antiseptic soap for correct handwashing. Considering the “hygiene habits of handlers,” the scores ranged from 8% to 38% adequacy. The lower scores were due to the absence of uniforms, the handling of food and money by the same person, and no periodic supervision of family farmers’ health. Regarding the questions about “production and process controls,” the scores ranged from 6% to 56% adequacy. The lower scores were due to the absence of temperature control for foodstuffs and inadequate storage. There were no labels or adequate packaging for ready-to-eat foods. Regarding the “implementation of the GMP manual”, the scores ranged from 0% to 59% adequacy. The lower scores were due to the absence of implemented GMP documents in almost all the examined public food markets and the absence of posters, banners, and flyers explaining hygienic food handling habits. Family farmers reported not having training about food handling or GMP before this study. 

Spearman’s correlation test (Figure 5) showed a very strong positive correlation between questions about “implementation of the GMP manual” and “production and process controls” (R = 0.95; *p* ≤ 0.05), as well as betweeb “production and process controls” and “hygiene habits of handlers” (R = 0.92; *p* ≤ 0.05). These results indicate that better production and process controls should be associated with GMP manual implementation and the adoption of satisfactory hygiene habits by food handlers.

A PCA was run with GMP data to order the public food markers regarding these food safety criteria (Figure 6), which showed that only two markets had minimal conditions of GMP with scores close to 50%. 

### 3.3. Training of Family Farmers and Assessment of Food Safety Practices Knowledge

The results of questionnaires about food safety practices given to family farmers before and after the application of the developed gamification educational strategy are shown in Table 1. This program involved game-based learning to share knowledge about food safety and hygiene practices.

There was no homogeneity between the answers (*p* ≤ 0.05) for knowledge about “prevention of foodborne diseases” and “food safe handling” before and after the application of the educational gamification strategy. These results indicate that this method promoted an increase in this knowledge area. However, there was homogeneity between the responses (*p* > 0.05) before and after the application of the educational gamification strategy for knowledge about “food inspection and storage,” indicating no change or increase in knowledge about it.

### 3.4. Microbiological Parameters of Foods Commercialized by Family Farmers in Public Food Markets

The results of the measured microbiological parameters of foods commercialized by family farmers before and after the application of the educational gamification strategy are shown in Table 2. There was a difference (*p* ≤ 0.05) between the microbiological counts found before and after the application of the educational gamification strategy for total coliforms, coagulase-positive staphylococci, and molds and yeasts. The maximum counts for total coliforms, coagulase-positive *Staphylococcus*, and molds and yeasts were higher than the limits allowed by Brazilian regulations [28], while the results for *E. coli, Salmonella* spp., *B. cereus*, and *L. monocytogenes* were adequate before and after the application of the educational gamification strategy.

The counts of total coliforms, coagulase-positive *Staphylococcus*, and molds and yeasts are indicative of the quality of food handling, the hygiene habits of handlers, and the hygiene of the environment where the food was produced (buildings and facilities). These results corroborate with those found in the questionnaires about food safety practices, which demonstrated an improvement in knowledge about foodborne diseases and food-safe handling, indicating the efficacy of the developed educational gamification strategy in increasing hygiene practices, reducing the transmission of foodborne diseases, and improving food-safe handling.

## 4. Discussion

This study verified only 75 family farmer groups commercializing in nine public food markets in the city of João Pessoa (Northeast region of Brazil), while studies assessing the socio-demographic profile of family farmers in other cities in Brazil identified 213 family farmer groups trading in each city, with an average of 21 different public food markets [14,33,34]. This shows the need to improve recommendations and promote actions envisaging better conditions and access to other family farmers in this region [34]. For Latin America, family farming plays a significant role in food security [35]. 

Regarding the products sold in these places, most were fruits, vegetables, tubers, and roots. Early studies showed that the Northeast and South Brazilian regions have the greatest diversification of food products commercialized by Brazilian smallholder agriculture. The greater diversification of these food products in Northeastern Brazil could be associated with the subsistence and food security of family farmers living in rural areas, since this region concentrates a significant part of the poorest Brazilian population [1,35,36]. The most important aspect of the diversity and application of this commercialization is the promotion of healthy feeding. The Brazilian guide to healthy eating advises buying foods from family farmer markets and giving preference to natural and local foods [35,36].

It is estimated that family farms represent 80% of all farms (17 million farms), occupy 35% of the cultivated land, and provide 27–67% of the total national production in Latin American countries. While covering a smaller land area than large-scale agriculture, family farms generate 57–77% of the total number of jobs in rural areas. In Brazil, family farming is largely responsible for producing beans, coffee, cacao, and horticultural crops [1,2,37,38]. These data reveal the importance of developing GMP training strategies for family farmers to increase the safe commercialization of their food products.

The smallholder farmers in Brazil tend to belong to associations and cooperatives that allow them to reach economies of scale in both input acquisition and output commercialization, such as street food or cooked/processed foods sold informally in marketplaces [1,2,37]. The main characteristics of these associations and cooperatives are self-management, results-sharing, and a horizontal structure, in which the family farmers have the autonomy to make their own decisions [8]. There was an increase in the number of cooperatives in Brazil, where their production increased from 1548 in 2010 to 1613 in 2018 [1,8,12]. Many of these groups seek to add economic value to their agricultural production through processing. However, the processing of the raw materials commonly takes place in an artisanal food processing plant, which may be a risk for the occurrence of foodborne diseases [8,12]. Therefore, the development of strategies to qualify family farmers and improve food safety practices is very important. Like this study, which shows the unsatisfactory GMP conditions, early investigations showed the lack of good hygienic practices for food preparation in street food in public markets and fairs [6,9,37,38,39]. These results indicate a need to promote effective training to improve the food safety practices of family farmers.

This study found that the most common street foods sold in family farmers’ public food markets were baking products (loaves, cookies, and cakes), cheeses, and fruit pulps. The commercialization of foods by family farmers offers a wide variety of foods, some of which are high risk, such as baking products, cheeses, and fruit pulps. Therefore, cross-contamination can occur due to improper food handling, contaminated equipment, and/or poor personal hygiene, especially when it involves ready-to-eat foods or street foods. Specific processing and handling measures, time, and temperature control must be implemented to ensure the safety of foods sold to consumers [9,37]. Although family farmers’ food markets are considered separate entities within the food business, they are often difficult to control, even being subject to official control. Key hurdles commonly include significant daily fluctuations in people, the turnover of different types of foods and other products, the production of a large amount of waste that needs to be properly disposed of, improper food handling, and exposure to unsatisfactory environmental conditions [9,38,39].

Other components found in this study that corroborate with previous investigations is the lack of infrastructure, inadequate working conditions, malfunctioning equipment, lack of potable water, and insufficient supervision, which are among the main barriers to achieving food safety conditions in various types of food establishments, including public markets and fairs, in developing countries [6,40,41]. Environmental, social, cultural, and belief factors may also affect the adoption of food safety practices by food handlers in their workplaces [18,42]. Indeed, the lack of adequate food safety training is among the most important issues in effectively implementing food safety practices in food marketplaces and food processing environments [43,44,45]. 

This study developed an education gamification strategy to deliver knowledge about food safety, aiming to improve the adoption of food safety practices by family farmers in public food markets in a city in Northeastern Brazil. Three training modules were developed, namely, (i) ways to prevent foodborne diseases, (ii) safe food handling, and (iii) safe food storage. These modules aimed to improve knowledge about personal hygiene, cross-contamination, environmental hygiene, food storage and process control, pest control, and cleaning and sanitation, respectively. Two commonly used approaches to training development are the Analyze, Design, Develop, Implement, and Evaluate (ADDIE) method and the Successive Approximation Model. Both approaches to training development consider learner needs and continually collect feedback to ensure that training is designed effectively for the learner to achieve learning objectives [7]. In this study, the developed strategies might be applied to family farmers who sold street foods in public food markets and fairs, as they are dynamic and easy strategies for everyone to understand and apply.

Food safety training for food handlers is often subdivided into specific behavioral constructs, such as personal hygiene, adequate cooking of foods, avoiding cross-contamination, keeping foods at safe temperatures, and avoiding food from unsafe sources, with the final purpose of preventing foodborne diseases [18,44]. These training programs should rely on food safety methodologies, such as GMP and good agricultural practices (GAP) to expand the knowledge of food handlers about food safety and foodborne illness prevention [6,35,46,47]. Studies with food handlers that had undergone previous training in food hygiene reported an increase in their knowledge and good food handling practices. On the other hand, using an educational gamification-based strategy should result in better concentration during class and better attitude changes by learners [48,49,50]. A meta-analysis of 47 studies that assessed the impact of food safety training on the knowledge, attitudes, and behaviors of food handlers reported that most training programs consisted of a face-to-face group training session that generally took less time of one day to complete [7]. In this study, the developed educational gamification strategy consisted of three sessions over a short period of time on different days.

The results of the microbiological parameters of foods commercialized by the family farmers in the public food markets showed decreases in the counts of total coliforms, coagulase-positive *Staphylococcus*, and molds and yeasts after the application of the developed educational gamification strategy. These results show that game-based learning can be an effective strategy to promote changes in food safety practices at family farmers’ food markets, with a direct impact on the improvement of microbiological parameters indicative of hygienic-sanitary conditions of foods commercialized in these places.

Previous studies have commonly shown unsatisfactory counts of microorganisms and/or microbial groups used to indicate the food hygienic-sanitary conditions in street food samples and the possible risks for the occurrence of foodborne disease outbreaks. These studies also indicate that food contamination should be reduced by avoiding common hazards in food production and monitoring the maintenance of GMP [37,51,52]. Studies with foods commercialized by family farmers have also reported unsatisfactory microbiological characteristics linked to not adopting GMP [12,13].

Cheeses are among the foods most involved in foodborne outbreaks, being incriminated in about 4% of all foodborne illnesses, which are more frequent in developing countries [51]. Coliforms, coagulase-positive staphylococci, and molds and yeasts are frequently found and considered indicators of food contamination by improper handling and a higher risk of foodborne disease [51,52]. Among the most important risk factors for the transmission of pathogens to foods are the poor hygiene of food handlers, insufficient cooking, lack of ideal temperature during storage, and cross-contamination between raw and ready-to-eat foods. All these risk factors can be minimized with appropriate training strategies for the target audience [7,52], which was the proposal approached in this study.

## 5. Conclusions

The results showed that public food markets where family farmers included in this study commercialized their products do not have adequate GMP conditions for food commercialization. However, there was a relation between the GMP manual implementation and the promotion of better production and process controls, as well as the adoption of satisfactory food safety practices by family farmers (food handlers) working in these markets, which was dependent on the application of the educational gamification food safety strategy developed in this study. This training was developed with a game-based learning strategy using three educational games to teach about foodborne illness, safe food handling, and safe storage and inspection, which was effective in increasing awareness about food safety practices. The developed educational gamification strategy also contributed to the improvement of microbiological parameters of foods commercialized by these family farmers. Finally, the educational gamification strategy developed in this study can be an effective tool for training in food safety practices for family farmers, helping to reduce the risk of foodborne diseases for consumers, considering the increasing interest in consuming food products from family farmers’ activity.

## Figures and Tables

**Table 1:** Response to questionnaires about food safety knowledge before and after the application of the developed educational gamification strategy.

Topics	Correctness Level of Responses		Chi-Square Test
	Before	After	*p* Value
Prevention of foodborne diseases 1. Pathogenic bacteria present in food can cause serious illness and can be prevented by proper cooking, cleaning, and sanitation practices? 2. Salmonellosis is a foodborne illness caused by a bacterium and can be prevented by washing hands before handling food? 3. Listeriosis is a serious infection caused by a bacterium and can be prevented by proper food storage and handling techniques? 4. *Bacillus cereus* can cause food poisoning and can be prevented by properly reheating precooked foods? 5. Cross-contamination occurs when bacteria from one food is transferred to another and can be prevented by keeping raw and ready-to-eat foods separated?	37 ± 2%	88 ± 1%	≤0.005
Safe food handling 1. It is recommended to wash hands before handling food and after using the cell phone or going to the bathroom? 2. Using a cell phone while handling food is a safe practice? 3. Singing or talking near food presents no risks for food safety? 4. Keeping nails short and without nail enamel can help prevent food contamination? 5. Handling money while handling food can be a risk to food safety?	67 ± 1%	92 ± 6%	≤0.005
Food inspection and storage 1. Is it necessary to always verify the shelf life of stored food? 2. Should frozen foods be stored below −18 °C? 3. Should chilled foods be kept below −4 °C? 4. Is it acceptable to store food on the floor? 5. Is it acceptable to store food together with cleaning materials?	75 ± 4%	95 ± 3%	>0.005

**Table 2:** Results of the microbiological analyses of foods commercialized at two selected public food markets before and after the application of the educational gamification strategy.

Analysis	Before			After			Friedman Test
	Median	Minimum	Maximum	Median	Minimum	Maximum	*p*
Total coliforms (CFU/g or mL)	2.40	<1	4.89	<1	<1	3.7	<0.001
*E. coli* (CFU/g or mL)	<1	<1	<1	<1	<1	<1	1.00
Coagulase-positive *Staphylococcus* (CFU/g or mL)	1.79	<1	4.69	<1	<1	3.30	0.027
*Salmonella* spp.	Absence	Absence	Absence	Absence	Absence	Absence	1.00
*B. cereus* (CFU/g or mL)	<1	<1	2.00	<1	<1	1.88	0.458
Molds and yeasts (CFU/g or mL)	2.70	<1	3.72	<1	<1	2.70	<0.001
*L. monocytogenes*	Absence	Absence	Absence	Absence	Absence	Absence	1.00

## Data Availability

Data is contained within the article.
